# LIHNCS - Lugol’s iodine in head and neck cancer surgery: a multicentre, randomised controlled trial assessing the effectiveness of Lugol’s iodine to assist excision of moderate dysplasia, severe dysplasia and carcinoma in situ at mucosal resection margins of oral and oropharyngeal squamous cell carcinoma: study protocol for a randomised controlled trial

**DOI:** 10.1186/1745-6215-14-310

**Published:** 2013-09-24

**Authors:** James A McCaul, James A Cymerman, Stuart Hislop, Chris McConkey, Jeremy McMahon, Hisham Mehanna, Richard Shaw, David N Sutton, Janet Dunn

**Affiliations:** 1 Bradford Institute for Health Research, Bradford Royal infirmary, Bradford, UK; 2 Crosshouse Hospital, Kilmarnock, Scotland, UK; 3 Clinical Trials Unit, University of Warwick, Coventry, UK; 4 Southern General Hospital, Glasgow, Scotland, UK; 5 Institute of Head and Neck Studies and Education, University of Birmingham, Birmingham, UK; 6 Head & Neck Cancer Research Group, University of Liverpool, Liverpool, UK

**Keywords:** Oral cavity, Oropharynx, Squamous cell carcinoma, Lugol’s iodine, Staining, Resection margins

## Abstract

**Background:**

Oral cavity and oropharynx cancer are increasing in incidence worldwide but survival outcomes have not significantly improved over the last three decades. The presence of dysplasia or carcinoma in situ at surgical margins following resection of squamous carcinoma of the mucosal surfaces of the head and neck has been shown to be associated with a higher incidence of local recurrence and reduced survival. While invasive carcinoma in mucosal surfaces can usually be distinguished from adjacent normal mucous membrane, pre-malignant disease is much less readily distinguished at operation. We describe a protocol for a randomised, controlled trial in which we will assess the effectiveness of Lugol’s iodine staining in allowing visualisation and excision of cancer margin dysplasia at time of primary surgery.

**Methods/Design:**

We will recruit 300 patients diagnosed with oral cavity or oropharynx squamous cell carcinoma. All participants will be planned for primary surgery with curative intent. After completion of baseline assessment participants will be randomised into either a standard surgical treatment arm or surgical treatment including Lugol’s iodine staining.

**Discussion:**

This paper describes the rationale and design of a unique trial in head and neck surgical oncology. If margin dysplasia visualisation with Lugol’s iodine allows complete excision of high-risk, pre-cancer mucosa at time of primary surgery, this may lead to a reduction in local recurrence and improved survival.

**Trial registration:**

Current Controlled Trials ISRCTN03712770.

## Background

The presence of dysplasia or carcinoma in situ at surgical margins following resection of carcinoma of the mucosal surfaces of the head and neck, has been shown to be associated with a higher incidence of local recurrence events [1,2].

While invasive carcinoma in mucosal surfaces can usually be distinguished from adjacent normal mucous membrane, pre-malignant disease is much less readily distinguished at operation. The mucosal surfaces of the head and neck are not unique in this respect. Attempts to improve the identification of early carcinoma and intraepithelial neoplastic change using adjunctive techniques are employed in the cervix, large bowel and oesophagus. These include acetic acid topical application, contact endoscopy, auto fluorescence and the use of Lugol’s iodine [3]. None of these has been subject of evaluation in a clinical trial setting in head and neck cancer.

Extensive literature exists regarding the use of Lugol’s iodine, including use as an aid to the identification of neoplasia of the squamous epithelium of the oesophagus. The structure of the mucosal surfaces of the mouth and oropharynx are very similar to the proximal oesophagus and the risk factors for neoplastic transformation at these sites are similar. Lugol’s iodine visualisation of dysplastic mucosa is employed in management of oesophageal disease [4-8]. There is, however, a very limited literature describing the use of Lugol’s iodine in the management of head and neck mucosal neoplasia and there are no reported clinical trials [8-10].

The majority of the oral cavity and oropharynx is covered by stratified, non-keratinising, glycogen-containing squamous epithelium. It is opaque, has multiple (15 to 20) layers of cells and appears pale pink in colour on visual examination. The intermediate and superficial layer cells contain glycogen in their cytoplasm. In contrast, dysplastic and invasive cancer cells contain little or no glycogen. This is thought to be due to the Warburg effect of increased cytosol glycolysis consequent to the genomic chaos seen in the cancer cell.

Iodine is glycophilic and forms tri-iodide molecules within the glycogen polymer spiral. This results in chocolate brown staining of normal non-keratinised oral and oropharyngeal mucosa. Areas of dysplasia and invasive cancer do not take up iodine as they lack glycogen and appear as pale coloured areas.

Heavily keratinised areas of the mouth such as the attached gingivae and hard palate do not avidly take up Lugol’s iodine. Respiratory mucosa also does not contain glycogen and does not stain. Thus detailed head and neck surgical anatomy knowledge is necessary in interpreting the information gained with Lugol’s staining. The major cancer sub-sites within the oral cavity and oropharynx are lined with glycogen containing and therefore Lugol’s iodine staining sites.

The presence of mucous on the surface interferes with the absorption of Lugol’s iodine. This can be minimised by the use of 1.25% carbocisteine solution prior to Lugol’s iodine.

Occasional mild adverse reactions have been reported [11], but our pilot and feasibility studies have shown that the use of mucosal epithelial staining with Lugol’s iodine is a safe technique, which is simply accomplished. With a short period of familiarisation it is readily adopted by experienced surgeons, is inexpensive, and seems likely to be effective. We believe that a trial demonstrating a clear benefit would have a substantial impact on clinical practice worldwide because of the simplicity of the technique and low cost.

## Methods/Design

### Study population

This study is designed as a prospective, multicentre, randomised controlled trial to assess the effectiveness of Lugol’s iodine in assisting excision of moderate dysplasia, severe dysplasia and carcinoma in situ at mucosal resection margins of oral and oropharyngeal squamous cell carcinoma. It fits into the standard practice of care for UK Head and Neck Cancer, and patients who meet the eligibility criteria (Table 1) and provide informed written consent will be enrolled. Detailed information on benefits and risks will be provided to study candidates to fully inform them of the process. Ethics approval has been received from NRES Committee Yorkshire & The Humber-Leeds East (REC reference: 10/H1306/29).

**Table 1:** Eligibility criteria

**Inclusion criteria**	**Exclusion criteria**
Provision of written informed consent	Previous surgery, chemotherapy or radiotherapy for head and neck cancer
Men and women aged >18 years	Allergy to iodine
Histologically proven squamous cell carcinoma of the oral cavity or oropharynx	Distant metastases (positive neck nodes are not an exclusion)
Planned for primary surgical treatment with curative intent	Nasal, nasopharyngeal or occult primary carcinoma
	Previous diagnosis of cancer in the past 5 years (except basal cell carcinoma or carcinoma of the cervix in situ).

### Randomisation and blinding

Patients will be randomly assigned in a 1:1 ratio to the Lugol’s or non-Lugol’s (control) group. Independent randomisation will be via telephone to the Warwick Medical School Clinical Trials Unit (WMSCTU). Computer-generated random permuted blocks will be used; stratification will be by treatment centre/surgeon, tumour site and T-stage. Treatment allocation will not be masked to the surgical team; however, the patient will not be informed of the treatment.

Data monitors, pathologists and external reviewers will be blinded to treatment allocations. The chief investigator, surgeons and other healthcare professionals will be blinded to all results.

The LIHNCS intervention is to be carried out in theatre during surgery.

**Figure 1:**
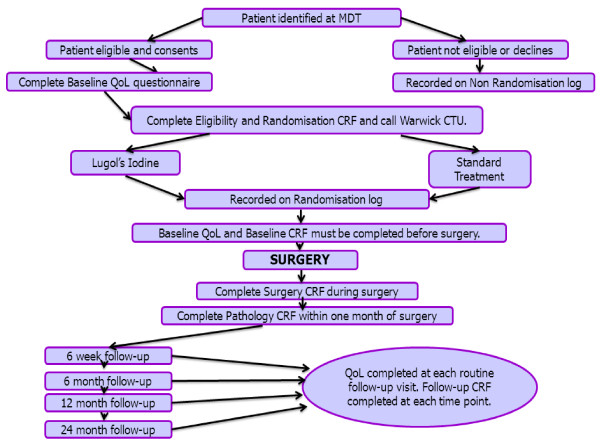
LIHNCS trial flowchart.

The trial follows the pathway as shown in Figure 1 below:

### Trial intervention and control

Patients in the intervention arm will have primary tumour stained with Lugol’s iodine at time of resection in the operating theatre. Those in the control arm will not have Lugol’s staining. All other intraoperative procedures will be the identical between trial and control arms.

The intraoperative standard operating procedure (SOP) covers application of Lugol’s iodine, tumour volume measured by volume displacement, and preparation of resected tumour for histopathology. It must be followed for all trial patients and is supplied below.

### Lugol’s iodine staining technique

Lugol’s Iodine staining to be performed under general anaesthetic using good retraction, light and assistance.

1. Tumour area irrigated with 0.9% saline.

2. Tumour area irrigated with 20 mL Carbocisteine (125 mg/5 mL).

3. Tumour area irrigated with 0.9% saline.

4. Tumour area irrigated with 20 mL of 1% Lugol’s iodine (4 mL elemental iodine/potassium iodide + 16 mL 0.9% saline) allowing a minimum of 30 s for staining to occur before aspirating excess fluid.

5. Tumour area irrigated with 0.9% saline.

6. Staining procedure may be repeated until staining is adequate.

7. Tumour resection planned to include a 1 cm oncologically safe margin and areas of non-stained mucosa where feasible.

Tumour volume to be measured by volume displacement

1. Standard 250 mL laboratory measuring cylinder filled with sufficient 0.9% saline to completely submerge the tumour sample that is to be measured.

2. Resected tumour added and tumour volume determined by volume displacement.

3. Record volume displaced on Surgery CRF form.

Preparation of resected tumour for histopathology

1. Primary specimen to be sutured to an appropriate platform (gamgee or corkboard) to allow appropriate display of resected specimen and accurate margin assessment.

2. Specimen to be sent to pathology as per standard site procedures.

### Assessments

Resected tumour samples will be analysed locally using recruiting site standard protocols. Slides and the accompanying pathology report are then sent to the trial coordinator where they are anonymised before being sent for central pathology review.

As part of a quality assurance process a central pathology review of all samples will be undertaken, performed by the trial pathologist. All external samples will be re-reported according to the Royal College of Pathologists minimum dataset for head and neck carcinoma histopathology.

In addition to this dataset, the central pathologist will report on the incidence of mild and moderate at mucosal resection margins in addition to severe dysplasia and carcinoma in situ. The minimum dataset from primary site pathology reporting will already report severe dysplasia and carcinoma in situ. Samples from the Bradford Teaching Hospitals NHS Foundation Trust will also be reviewed, as the purpose of the quality assurance of a national spread of samples is to standardise all reports against the reviewing pathologist.

### Follow-up protocol

The trial duration is expected to take 4 years in total.

Patients will be recruited over a 2-year period and will be followed up for a minimum of 2 years.

Quality of Life (QoL) questionnaires and CRFs will be completed at set time points (Figure 1) when the patient attends for their routine clinic appointments. QoL will be measured using the EORTC QLQ-30 and H&N disease specific EORTC QLQ-35 module questionnaires. Swallowing-related QoL will be measured using the MD Anderson Dysphagia Inventory (MDADI) questionnaire.

It is possible that since more tumours are likely to be removed in the Lugol’s iodine arm, there may be an effect on functionality and quality of life.

At each time point, each domain of each questionnaire will be compared between groups by ACNOVA, that is, linear regression with the follow-up score as the dependent variable, group as the predictor and baseline score as the covariate. Differences between groups will be calculated along with 95% confidence intervals (CI) and *P* values.

### Safety and adverse event reporting

In accordance with Good Clinical Practice for Research guidelines the occurrence of adverse events will be monitored carefully and recorded in detail during the course of the clinical trial.

If during that time a serious adverse event (SAE) is thought to be related to involvement in the trial and is unexpected the trial office will report this event to the Multicentre Research Ethics Committee (MREC).

The Principal Investigator in each centre must report any SAEs to the trial coordinator within 24 h of awareness of it. The SAE form will be completed and faxed to the trial coordinator who will then liaise with the Chief Investigator to compile all necessary information. The trial office will be responsible for reporting SAEs to the Sponsor and MREC within the required timelines.

### Study endpoints

The primary outcome measure will be rate of surface dysplasia, carcinoma in situ or carcinoma at surface mucosal margins in the Lugol’s-treated group *versus* gold standard management control arm. Secondary endpoints are detailed in Table 2.

**Table 2:** Secondary outcomes

	**Secondary outcome measures:**
1.	Acceptability of the technique to surgeons carrying out surgery for oral and oropharyngeal carcinoma.
2.	Effect of Lugol’s technique on any further treatment carried out (radiotherapy, chemoradiotherapy or further surgery).
3.	Estimate of the two-year locoregional recurrence rates in each group.
4.	Mean and range of volume of tissue removed by each method.
5.	Safety of the technique.
6.	Assessment of quality of life changes using EORTC QLQ-30 and EORTC-35 H&N questionnaires, and the MD Anderson Dysphagia Inventory (MDADI) questionnaire
7.	Overall survival

### Secondary outcomes

The primary endpoint of the presence of dysplasia in the resection margins will be compared using Pearson’s chi-squared test when all 300 patients have been recruited. There will be planned sub-group analysis by T stage to assess the effect of tumour size. T1 and T2 primary cancers will be compared with T3 and T4 cancers. There will be a pre-planned interim analysis when the first 164 patients have been recruited in order to assess whether the assumptions made in the power calculations hold true.

The total sample size is to be 300 patients, with an estimated recruitment rate of 15 patients per site per year over a 2-year period.

### Sample size calculation

In an initial study of two series of 50 consecutive patients having resection of oral or oropharyngeal SCC 32% in the standard group and 4% in the Lugol’s iodine group had dysplasia, carcinoma in situ or invasive SCC at a surgical margin (McMahon et al., 2012).

The primary outcome measure is rate of dysplasia of any grade at excision margins. We calculated that to detect a reduction from 30% in the control group (as in the initial cohort study) to 10% in the Lugol’s iodine group with 90% power at a two-sided significance level of 0.05, the number of patients required in each group is 82. The rate for the control arm may be a little lower. If it is 20% then a similar proportionate reduction to 7% would require 144 in each group. We propose to randomise 150 to each group, 300 patients in total. However, should the proportion of dysplasia positive cases change from 17% (control) to 4% in the Lugol’s group, then the power calculation would require 100 patients in each group for 80% power and 130 patients in each group for 90% power. We therefore aim to continue planning for recruitment of 300 patients overall and 150 in each group.

The recurrence rate in the dysplasia group is estimated at 33% [1]. To detect a difference of 25% in 20% of the patients (that is, those who convert from involved margins to non-involved as a result of the intervention), it is necessary to detect a difference of 5% overall and a much larger study would be needed (even with alpha = 0.1 and one-sided test over 1,200 patients would be needed).

Instead we will estimate a 95% CI for the hazard ratio.

## Discussion

The Head and Neck Cancer research literature contains very few well-designed trials and there are no published trials in surgical oncology regarding primary surgery for cancer at this site.

Our cohort study data show a reduction in margin dysplasia using Lugol’s iodine at cancer resection from 32% for control to 4% for intervention groups [12]. Our feasibility study showed very high patient acceptance rates (unpublished data) and we are very optimistic that this trial will recruit participants.

We are very fortunate to have the support of the CTAAC of Cancer Research UK and hence NIHR portfolio status for this study. This provides support from the Clinical Research Network (CRN) and has enabled LIHNCS to reach a wider audience and undoubtedly helped in enhancing recruitment to this trial to date.

## Trial status

Open and recruiting.

## Abbreviations

CTAAC: Clinical trials awards and advisory committee; CRF: Case report form; CRN: Clinical research network; CTIMP: Clinical trial of an investigational medicinal product; CTU: Clinical trials unit; DSMC: Data safety and monitoring committee; DMEC: Data monitoring and ethics committee; ECOG: Eastern cooperative oncology group; GCP: Good clinical practice; ICH: International conference on harmonisation; LIHNCS: Lugol’s iodine in head and neck cancer surgery; MDADI: MD Anderson dysphagia inventory; MDT: Multidisciplinary team; MREC: Multicentre research ethics committee; NIHR: National institute for health research; QoL: Quality of life; R&D: Research and development; SAE: Serious adverse event; SOP: Standard operating procedure; TSC: Trial steering committee; WMSCTU: Warwick medical school clinical trials unit.

## Competing interests

The authors declare that they have no competing interests.

## Authors’ contributions

JMcC is the Chief Investigator of the LIHNCS Trial and is a member of the trial management committee & the TSC. JAC is a member of the trial management committee, and is the Head & Neck Surgery Research Fellow at the Bradford Institute for Health Research. SH, JM, HM, RS and DNS are Co-Investigators and have roles in the initial cohort studies leading to the LIHNCS trial and the trial design. CMcC is the trial statistician. JD is the Warwick University CTU Lead for the trial. All authors edited and approved the final manuscript.
